# In Silico Modeling of Myelin Oligodendrocyte Glycoprotein Disulfide Bond Reduction by Phosphine-Borane Complexes

**DOI:** 10.3390/ph17111417

**Published:** 2024-10-23

**Authors:** Raheem Remtulla, Sanjoy Kumar Das, Leonard A. Levin

**Affiliations:** 1 Department of Ophthalmology and Visual Sciences, McGill University, Montreal, QC H3A 0G4, Canada; raheem.remtulla@mail.mcgill.ca; 2 Division of Experimental Medicine, McGill University, Montreal, QC H3A 0G4, Canada; sanjoy_das@videotron.ca; 3 Drug Discovery Core, Research Institute of the McGill University Health Centre, Montreal, QC H4A 3J1, Canada; 4 Department of Neurology and Neurosurgery, McGill University, Montreal, QC H3A 0G4, Canada

**Keywords:** phosphine-borane complexes, myelin oligodendrocyte glycoprotein, disulfide bonds, bond cleaving, optic neuritis, neurodegeneration

## Abstract

Background: Neurodegenerative diseases can cause vision loss by damaging retinal ganglion cells in the optic nerve. Novel phosphine-borane compounds (PBs) can protect these cells from oxidative stress via the reduction of disulfide bonds. However, the specific targets of these compounds are unknown. Proteomic evidence suggests that myelin oligodendrocyte glycoprotein (MOG) is a potential target. MOG is of significant interest due to its role in anti-MOG optic neuritis syndrome. Methods: We used in silico modeling to explore the structural consequences of cleaving the extracellular domain MOG disulfide bond, both in isolation and in complex with anti-MOG antibodies. The potential binding of PBs to this bond was examined using molecular docking. Results: Cleaving the disulfide bond of MOG altered the structure of MOG dimers and reduced their energetic favorability by 46.13 kcal/mol. The energy profiles of anti-MOG antibody complexes were less favorable when the disulfide bond of MOG was reduced in the monomeric state by 55.21 kcal/mol, but the reverse was true in the dimeric state. PBs exhibited reducing capabilities with the MOG extracellular disulfide bond, with this best-scoring compound binding with an energy of −28.54 kcal/mol to the MOG monomer and −24.97 kcal/mol to the MOG dimer. Conclusions: These findings suggest that PBs can affect the structure of MOG dimers and the formation of antibody complexes by reducing the MOG disulfide bond. Structural changes in MOG could have implications for neurodegenerative diseases and anti-MOG syndrome.

## 1. Introduction

Vision loss is a frequent result of neurodegenerative diseases affecting the optic nerve. Typical presentations include a loss of visual acuity, visual field defects, and dyschromatopsia [1,2]. The mechanism of action of these diseases is usually due to damage to retinal ganglion cell (RGC) axons within the optic nerve [1,2]. Damage to RGC axons induces RGC soma death through a variety of mechanisms. One such mechanism is via signaling from reactive oxygen species (ROS), particularly superoxide species [2,3,4,5]. Intracellular superoxide bursts have been identified as a critical event in RGC death and may act as a pre-apoptotic biomarker [5]. Evidence for this mechanism includes studies demonstrating increased levels of superoxide species in RGCs after injury, and the specified imaging of superoxide demonstrated that the level of superoxide increases before other markers for apoptosis in damaged RGCs [2,3,4]. Furthermore, superoxide scavengers that decrease superoxide signals have been able to prevent cell death in traumatic optic neuropathy models [2,3,4,5]. These studies suggest that superoxide species are upstream signals for RGC death [2,3,4,5]. Fortunately, disulfide reducing agents have been able to protect RGCs from ROS signal-related injuries [2,3,4,5]. Based on the neuroprotective properties of disulfide reducing agents, both in vitro and in vivo, we hypothesized that the mechanism by which ROS signal axonal injury is through disulfide formation [6,7]. Highly oxidative environments increase the formation of disulfide bonds between cysteine side chains and cause cellular damage [6,7].

Phosphine-borane compounds are a novel class of compounds recognized for their reactive organophosphine group and protective borane group, which have demonstrated promise in the field of neuroprotection [7,8,9]. In vitro and in vivo studies demonstrated that these compounds protect RGCs from oxidative stress [7,8,9]. Studies using triphenyl phosphine and aryl disulfides have suggested that phosphorus atoms in phosphine-borane molecules act as a nucleophile in an S_N_2 nucleophilic substitution reaction in disulfide bond cleavage [7,8,9,10]. However, the exact target of novel phosphine-borane compounds has not been determined. Oxidative isotope-coded affinity tag data from our laboratory was used to identify one of the protein targets for the bis(3-propionic acid methyl ester) phenylphosphine borane complex (PB1) as myelin oligodendrocyte glycoprotein (MOG), with consistent disulfide bond cleavage.

MOG is a clinically relevant protein for neurodegeneration because of anti-MOG-related neurologic disease, which partially overlaps with multiple sclerosis. Anti-MOG syndromes result from damage to MOG [11,12], which is expressed on oligodendrocyte cell surfaces and on the outermost surface of myelin sheaths [11]. Although early studies found that 41% of multiple sclerosis (MS) patients had anti-MOG antibodies in serum, as did patients with non-MS neurologic disease and healthy patients [12,13,14,15,16,17], it is now known that anti-MOG antibodies are rare in patients with typical MS phenotypes [11] and that anti-MOG syndromes are clinically distinct entities from MS. Unlike MS, anti-MOG patients present more frequently with bilateral optic neuropathy, steroid dependence, and a lower female predominance [11,18,19]. Interestingly, the clinical presentations of anti-MOG syndrome differ between adults and children [11]. In children, the most common presenting symptom is acute disseminated encephalomyelitis (36%), whereas the most common presentation for anti-MOG syndromes in adults is optic neuritis (88%) [11,19,20]. Unlike in MS, bilateral optic neuritis is common, with one study finding 42% of anti-MOG syndromes had bilateral optic neuritis and 31% had unilateral optic neuritis [19,20]. Also unlike MS, optic disc swelling is seen in the majority of patients. Anti-MOG optic neuritis is comparatively severe, with visual acuity worse than 20/200 [19,20]. Anti-MOG optic neuritis is highly steroid-responsive, with rapid improvement in symptoms after intravenous methyprednisone. Unfortunately, the cessation of steroids often results in relapses of optic neuritis, and patients often must continue long-term steroid-sparing immunosuppressants to prevent recurrences. Although immunosuppression can prevent recurrent optic neuritis, it can be associated with systemic side effects, including recurrent infections, lymphocytopenia, hepatotoxicity, nephrotoxicity, and cardiac disease. Patients on immunosuppression have to be monitored for these adverse effects [11,18].

**Figure 1:**
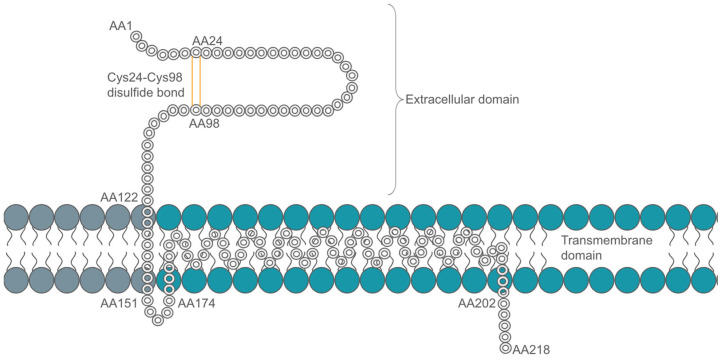
Simplified schematic of MOG based on Kroepfl et al. proposed model [28,29].

MOG is a minor myelin component (~0.05% of all myelin proteins), which is exclusively expressed in the central nervous system (CNS) of mammals, on the outermost lamella of myelin sheaths and oligodendrocyte processes [21,22]. MOG is a type I integral transmembrane protein with a single extracellular Ig variable domain [22,23,24]. A schematic of the MOG structure is presented in Figure 1. There are 245 amino acids, of which the first 126 are extracellular [23,25,26,27]. The protein is relatively small, with dimensions of 40 × 35 × 30 Å [23,25,26,27] and a ß sandwich domain with one antiparallel ß sheet, with the N and C terminals on opposite ends of the monomer. Like many Ig-V domains, MOG has core packing residues, with a disulfide bond between Cys24 and Cys98 and a buried salt bridge between Arg68 and Asp92. There are three loops at the end, distal to the membrane, including an F-G strand that has been implicated in autoimmunity. The structure also has a significant strip of electronegative charge that runs about half the length of the molecule [25].

Native MOG exists as a mixture of monomeric and dimeric species [25]. The extracellular domain has the ability to form a homodimer in situ within the CNS [30]. A crystal structure of the dimer was described by Clements et al. The dimer forms an antiparallel head-to-tail dimer. The dimer interface involves interactions between residues 8–11 interacting with opposing residues 95–112, and residues 40–46 and opposing residues 40–46. The interaction is stabilized by 10 hydrogen bonding and van der Waals interactions [25]. Native MOG has been identified in both monomeric and dimeric forms both in and outside the central nervous system [22,30,31]. The amino acids 35–55 are buried in dimer formation, and have also been implicated in autoimmunity to MOG. Given that gel filtration identified more MOG existing as monomers than as dimers, one could infer that the dimer interface is weak and favors monomer formation over dimer formation [25].

In order to determine the nature of antibody binding to MOG, the crystal structure extracellular domain of MOG from rat was docked with the chimeric antigen-binding fragment FAb of the demyelinating MOG specific monoclonal antibody 8-18C5 [21,31]. The MOG-FAb complex showed that the antibody bound to the upper membrane distal surface of MOG, binding to three loops located at the membrane distal side of MOG, with the major contribution made by the FG loop. The FG loop formed the major interactions of the complex, and the FG loop includes amino acids 101–108 which became completely buried within the 8-18C5 antibody [21]. The sequence I of AA 101–108 is unique to MOG, which is only in the CNS. Therefore, the body does not develop immune tolerance to that sequence.

Other encephalitogenic epitopes of MOG include AA 1–22, AA 35–55 and AA 92–105 [25,32,33,34]. MOG AA 35–55 is of particular interest, being able to cause inflammatory and demyelinating immune responses [32,33,34]. Interestingly, the epitopes of mAb 8-18C5 and other demyelinating mAbs to MOG are conformation-dependent, and linear epitopes to MOG do not induce widespread demyelination [35]. This suggests that disruption in MOG’s baseline structure may prevent autoimmunity. Both the monomer and the dimer species have been recognized by 8-18C5 antibodies [25].

Using in silico methodologies and molecular docking, we tested whether phosphine-borane compounds cleave the disulfide bond in the extracellular domain of MOG. Such data would be valuable in determining the mechanism of these novel compounds, provide insight into the mechanism of anti-MOG-associated optic neuritis, and identify a potential new target of neuroprotection for demyelinating disease.

## 2. Results

### 2.1. Preprocessing of MOG Structure

An X-ray diffraction of the monomer structure of MOG from Breithaupt et al. [21] was accessed as a PBD file on the RCSB protein data bank (PBD 1PKO; resolution 1.45 Å) and imported directly into Schrödinger. The PBD validation from the RCSB demonstrated that the protein was an appropriate structure compared to other X-ray diffraction structures on the database, with the only metric below average being real space R-value Z-score RSRZ outliers. Both the Ramachandran and sidechain outliers were appropriate. A protein reliability report was generated after importing it to Schrödinger. Only 2 of the 22 metrics evaluated were flagged. The first was missing atoms, where only two amino acids were involved. The second included three water molecules that did not form hydrogen bonds with the protein structure. These issues were managed with the protein preparation tool and the resultant structure utilized as the ground truth for the monomer structure with the Cys24-Cys98 disulfide bond intact (MOG-DI).

Breithaupt et al. also developed an X-ray diffraction of the MOG dimer in complex with anti-MOG antibodies (PBD 1PKQ; resolution 3 Å) [21]. However, the PBD validation from the RCSB suggested that the structure was relatively poor, with no metrics of structure quality above the 50th percentile for all PBD X-ray diffraction structures other than RSRZ outliers. Seven of the other 22 metrics evaluated in the Schrödinger protein reliability report for 1PKQ were out of the appropriate range. The most significant out-of-range metric was steric clashing, of which there were 89 clashes between amino acids on the structure. Finally, the resolution of the structure was considered unacceptable. These structures were used in antibody docking; however, the lower quality of these structures should be noted.

### 2.2. Impact of Disulfide Bond on MOG Monomer Structure

The structure of MOG-DI was evaluated in the workspace and the disulfide bond between Cys24 and Cys98 cleaved manually. The post-cleaved MOG structure was processed with the protein preparation wizard without automatic disulfide formation. Minimization was performed and the resultant structure named MOG-DR (MOG with disulfide bond reduced).

MOG-DI was then compared to MOG-DR. There was little difference between the two, with only a 0.19 Å root mean squared deviation (RMSD) between the FG loops on MOG-DI and MOG-DR. There was a 55.21 kcal/mol difference in minimized total complex energy favoring MOG-DR monomers over MOG-DI monomers. Table 1 demonstrates the complex energies of all structures in the proteomic analysis.

### 2.3. Impact of Disulfide Bond on MOG Dimer Structure

**Figure 2:**
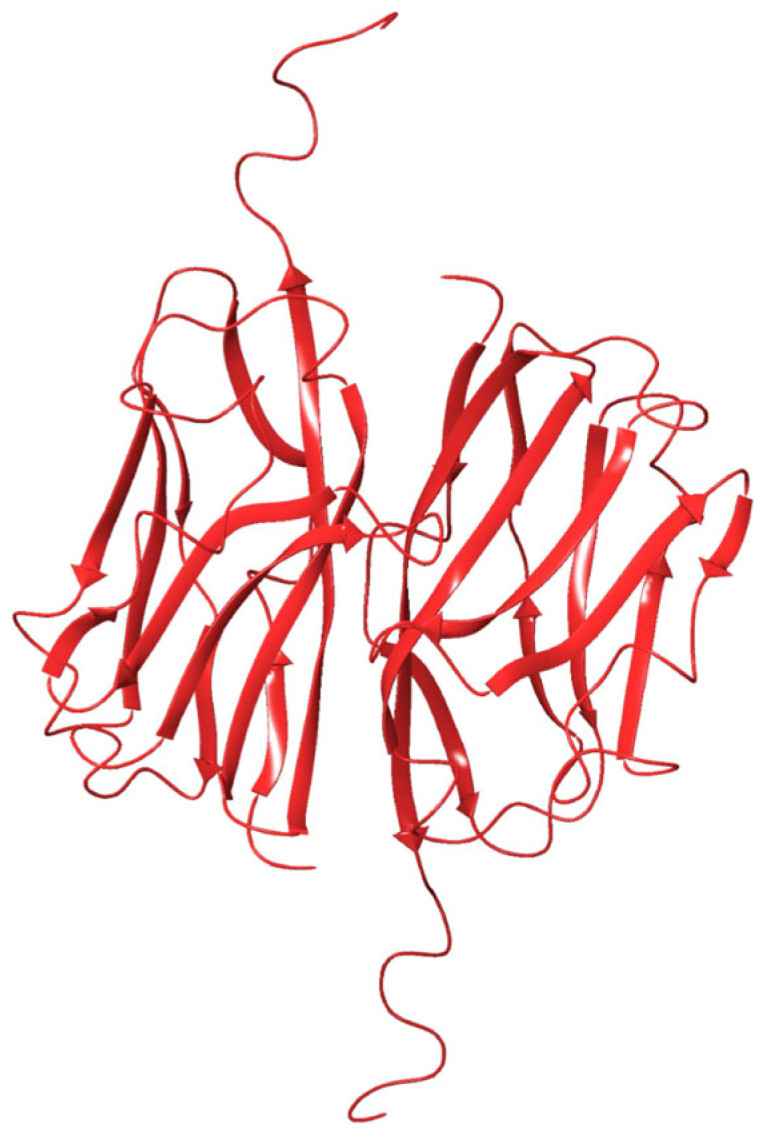
MOG dimer protein ribbon structure of Schrödinger protein–protein docking of extracellular MOG monomers with disulfide bond intact (PBD 1PKO).

Using MOG-DI, protein–protein docking was performed to generate a computational model of the MOG-DI dimer (Figure 2). This resulted in a total of 22 poses, of which one fit the description of the dimer described by Clements et al. [25], with a total complex energy of −1218.89 kcal/mol and an RMSD of 0.05 (Table 1). The dimer interface had a total interface interaction of 1023 Å^2^ and 1037 Å^2^. There were also 8 salt bridges and 15 hydrogen bonds identified at the interface (Table 2).

**Figure 3:**
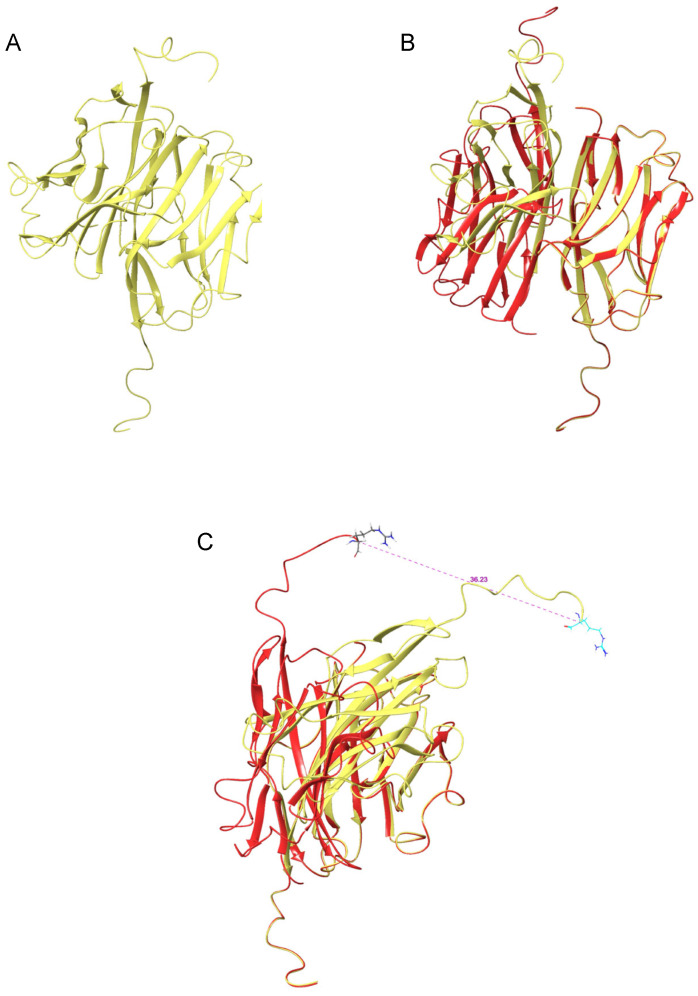
(**A**) MOG dimer protein ribbon structure of Schrödinger protein–protein docking of extracellular MOG monomers with disulfide bond cleaved pre dimer formation (PBD 1PKO). (**B**) Overlapping structures of MOG dimer protein ribbon structure with disulfide bond intact (RED) and MOG dimer protein ribbon structure with disulfide bond cleaved (YELLOW-GREEN) from Schrödinger protein–protein docking (**C**) Lateral view of overlapping structures of MOG dimer protein ribbon structure with disulfide bond intact (RED) and MOG dimer protein ribbon structure with disulfide bond cleaved (YELLOW-GREEN) from Schrödinger protein–protein docking. Measurement between arginine 126 on MOG dimer protein ribbon structure with disulfide bond intact and MOG dimer protein ribbon structure with disulfide bond cleaved in Ångstrom units.

The process of dimer formation with protein–protein docking was repeated using MOG-DR. A total of 21 poses resulted, of which none were in keeping with the structure suggested by Clements et al. [25], with one structure closely similar to the known dimer between MOG monomers (Figure 3). The latter structure had a complex energy of −1172.76 kcal/mol and an RMSD of 0.10 (Table 1). There were no salt bridges at the interface and only six hydrogen bonds (Table 2). There was significant rotation of the DR monomers in the MOG-DR dimer structure compared to the MOG-DI dimer, with Arg126 displaced by 36.23 Å in the MOG-DR dimer compared to the MOG-DI dimer. Furthermore, the antigenic chain was shifted, with an RMSD of 11.95 Å between the MOG-DI dimer and the MOG-DR dimer [25].

There was a 46.13 kcal/mol difference in complex energies favoring the MOG-DI dimer over the MOG-DR dimer (Table 1). This was further exemplified by the presence of a greater number of residues, a larger interface area, and a greater number of salt bridges and hydrogen bonds. This suggests that the Cys24–Cys98 disulfide bond of the MOG monomer is important for MOG dimer formation.

### 2.4. Impact of Disulfide on Interactions Between Anti-MOG Antibody and MOG Monomer

Given the role of the disulfide on MOG dimer formation, the nature of the binding of anti-MOG antibodies to MOG was explored. The MOG monomer in complex with the antibody [21] (PBD 1PKQ) was processed by isolating the antibody from this structure and minimizing it to its lowest energy state using the protein preparation tool. After minimization, the heavy chain from the antibody isolated from 1PKQ was docked with the protein–protein docking tool to three structures: the monomer remnant from 1PKQ, the MOG-DI monomer, and the MOG-DR monomer.

First, the top 30 poses resulting from docking the MOG monomer remnant (derived from 1PKQ) to the isolated heavy chain of the anti-MOG antibody (derived from 1PKQ) were analyzed, one of which matched the monomer–heavy chain antibody complex represented by 1PKQ. This pose, being closest in structure to 1PKQ, had a total complex energy of −1276.54 kcal/mol (Table 1). The RMSD between the MOG FG loop in the docked results and the FG loop in 1PKQ was 1.97 Å. This suggests that despite the lower resolution of 1PKQ, the protein–protein docking tool was able to redemonstrate the interactions between MOG and anti-MOG antibodies.

Second, the top 30 poses resulting from the docking of the MOG-DI monomer (derived from 1PKO) to the isolated heavy chain of the anti-MOG antibody (derived from 1PKQ) were analyzed, one of which matched the monomer–heavy chain antibody complex represented by 1PKQ. The pose had a total complex energy of −1332.73 kcal/mol and an RMSD of 0.05 (Table 1). The presented structure had 24 residues at the antibody–monomer interface, a total interface area of 1169 Å^2^, 3 salt bridges, and 9 hydrogen bonds (Table 2). The modeled antibody complex was compared with 1PKQ. There was an insignificant difference in the location of antigenic chain binding to the antibody between the two structures, with an RMSD of 1.19 Å between the backbones of the FG loops of the two structures.

Third, the MOG-DR monomer from 1PKO was docked to the isolated heavy chain of the anti-MOG antibody from 1PKQ, and the top 30 poses analyzed. There were significant differences between the complex of the MOG-DR monomer and the antibody vs. the monomer–heavy chain antibody complex represented by 1PKQ. Similarly, there were significant differences between the complex of the MOG-DR monomer and the antibody vs. the complex of the MOG-DI monomer and the antibody. The MOG DR monomer–antibody complex that was closest in structure to the 1PKQ ground truth structure had a total complex energy of −1333.79 kcal/mol and an RMSD of 0.05 (Table 1). There were 21 residues at the MOG-DR monomer–antibody interface, a total interface area of 1116 Å^2^, 3 salt bridges, and 5 hydrogen bonds (Table 2). The RMSD between the FG loops of the MOG-DR monomer–antibody complex and the complex of 1PKQ was 3.84 Å. In comparison, the RMSD between the FG loops of the MOG-DR monomer–antibody complex and the MOG-DI monomer–antibody complex was 3.02 Å.

There was only a 1.06 kcal/mol difference favoring the MOG-DR monomer–antibody complex over the MOG-DI monomer–antibody complex. The MOG-DR monomer had a 55.21 kcal/mol lower complex energy than the MOG-DI counterpart (Table 1). When considering the intrinsic difference in monomer–antibody complex energies, there is an approximately 54 kcal/mol complex energy difference favoring the MOG-DI monomer–antibody complex formation vs. the reduced form. This is further exemplified by increased interface residues, interface area, salt bridges, and hydrogen bonds with the MOG-DI monomer–antibody complex structure.

### 2.5. Impact of Disulfide on Interactions Between Anti-MOG Antibody and MOG Dimer

The analyses above established that the Cys24-Cys98 disulfide bond played a pertinent role in forming MOG antibody complexes. To explore the same process with the dimeric form of MOG, dimers of MOG-DI monomers and MOG-DR monomers were formed using the protein–protein docking tool, and then docked to the heavy chain antibody isolated from 1PKQ, as described previously.

**Figure 4:**
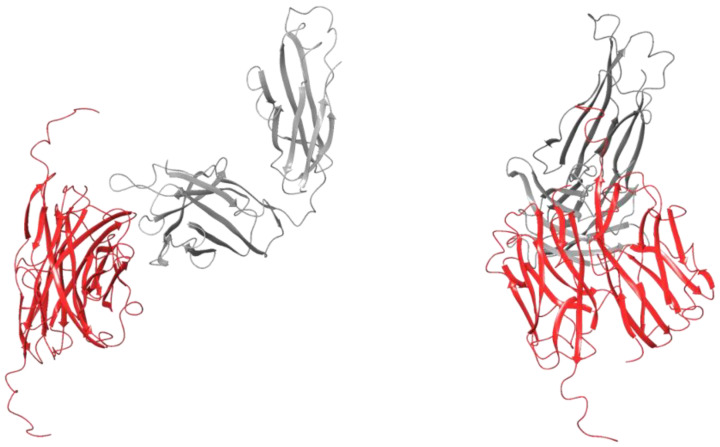
Protein ribbon structure of Schrödinger protein–protein docking-generated MOG dimer presented in the red structure (PBD 1PKO) with disulfide bond intact in complex with anti-MOG antibody presented in the grey structure(PBD 1PKQ). Two lateral orientations of the protein interaction are depicted.

Protein–protein docking between the MOG-DI dimer and the heavy chain of 1PKQ resulted in 30 poses. The X-ray crystallography of the dimer–antibody complex was not available, and therefore the structure analyzed was the one with the most structural similarity to the monomer–antibody complex (Figure 4). This structure had a complex energy of −2029.64 kcal/mol and an RMSD of 0.05 Å (Table 1). There were 23 residues at the interface, 3 salt bridges, and 7 hydrogen bonds, and the area of the interface was 1356 Å^2^ (Table 2). The RMSD between the FG loops on 1PKQ and the MOG-DI dimer–antibody complex was 5.55 Å. The RMSD between the FG loops on the MOG-DI dimer–antibody complex and the MOG-DI monomer–antibody complex was 4.92 Å.

**Figure 5:**
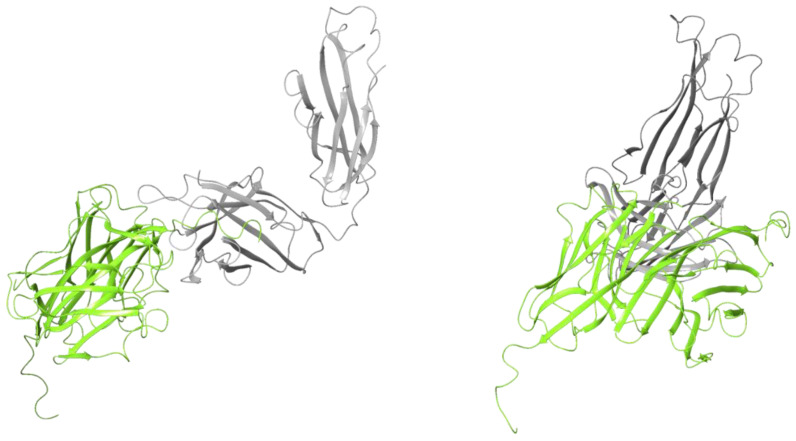
Protein ribbon structure of Schrödinger protein–protein docking-generated MOG dimer presented in the yellow-green structure (PBD 1PKO) with disulfide bond cleaved pre dimer formation in complex with anti-MOG antibody presented in the grey structure (PBD 1PKQ). Two lateral orientations of the protein interaction are depicted.

**Figure 6:**
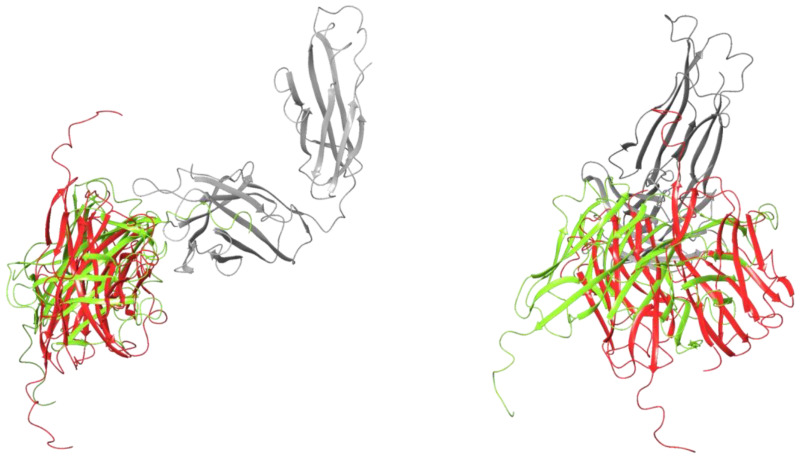
Overlapping structures of MOG dimer with disulfide bond intact presented in the red structure in complex with anti-MOG antibody presented in the grey structure (PBD 1PKQ), and MOG dimer with disulfide bond cleaved pre dimer formation presented in the yellow-green structure in complex with anti-MOG antibody presented in the grey structure (PBD 1PKQ). Two lateral orientations of the protein interaction are depicted.

Similarly, the MOG-DR dimer was docked to the heavy chain of the antibody from 1PKQ, resulting in 30 displayed poses. None of the presented structures were similar to the MOG-DI dimer–antibody complex. The structure that was most similar to the MOG-DI dimer–antibody complex had a total energy of −2032.65 kcal/mol and an RMSD of 0.05 Å (Table 1, Figure 5). There were 34 residues at the interface, one 1 salt bridge, and 7 hydrogen bonds, and the area of the interface was 1609 Å^2^ (Table 2). The RMSD between the FG loops on the MOG-DR dimer–antibody complex and the MOG-DI dimer–antibody complex was 4.71 Å, and the distance between the Arg126 on the MOG-DI–antibody complex and that of the MOG-DR dimer–antibody complex was 36.94 Å. The RMSD between the FG loops on the intact 1PKQ complex and the MOG-DR dimer–antibody complex was 6.87 Å, while the RMSD between the FG loops on the MOG-DR dimer–antibody complex and the MOG-DI monomer–antibody complex was 6.17 Å (Figure 6).

The dimer–antibody complex models demonstrated that there was a 3.01 kcal/mol complex energy difference favoring dimer–antibody complex formation when the Cys24-Cys98 disulfide was reduced, compared to a 46.13 kcal/mol difference in energy favoring dimer formation when the disulfide was intact (Table 1). These findings suggest a more favorable energy for a dimer–antibody complex with the disulfide bond reduced vs. the disulfide bond intact. This was further exemplified by a greater number of interface residues (34 vs. 23) and a larger interface area (1609 vs. 1356) when the disulfide bond was reduced, although more salt bridges were present (1 vs. 3) when the disulfide bond was intact (Table 2).

### 2.6. Covalent Docking of Phosphine-Borane Compounds to the MOG Monomer Disulfide

**Figure 7:**
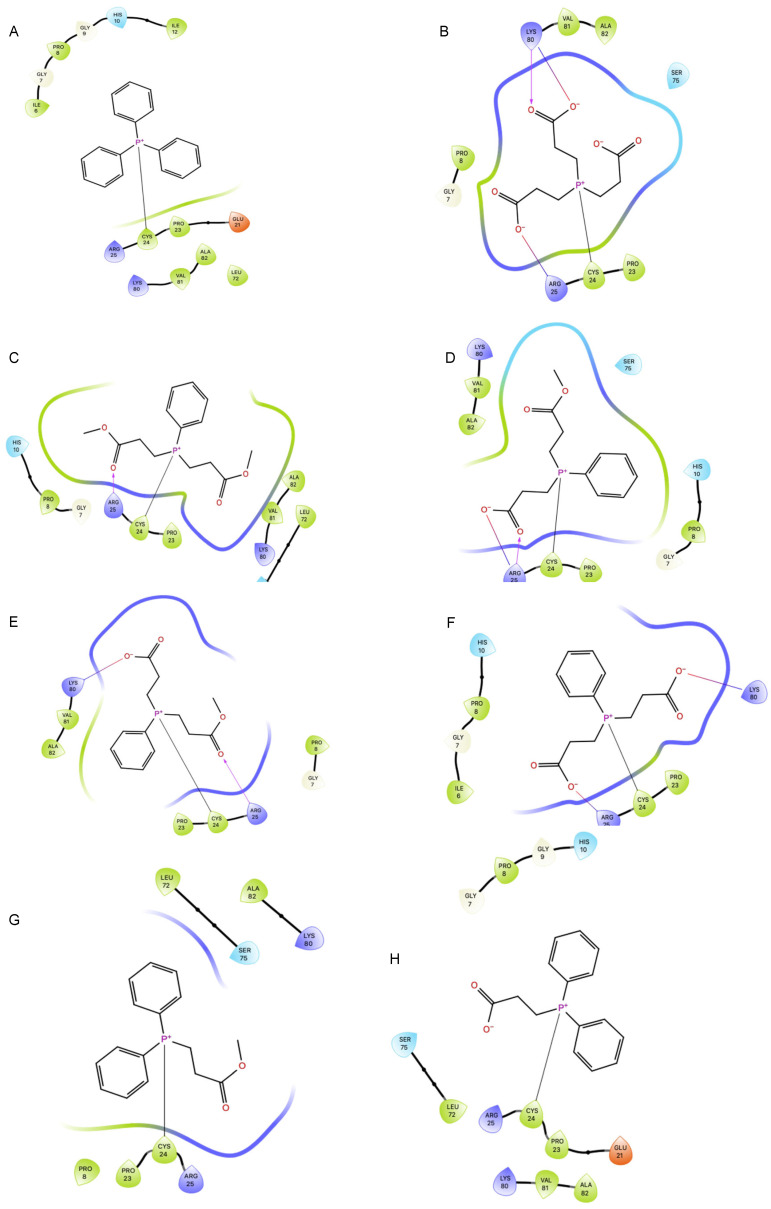
(**A**) Ligand interaction diagram of covalent docking of triphenylphosphine to cystine 24 on myelin oligodendrocyte glycoprotein. (**B**) Ligand interaction diagram of covalent docking of tris(2-carboxyethyl)phosphine to cystine 24 on myelin oligodendrocyte glycoprotein. (**C**) Ligand interaction diagram of covalent docking of P1 to cystine 24 on myelin oligodendrocyte glycoprotein. (**D**) Ligand interaction diagram of covalent docking of P1 monoacid R to cystine 24 on myelin oligodendrocyte glycoprotein. (**E**) Ligand interaction diagram of covalent docking of P1-monoacid S to cystine 24 on myelin oligodendrocyte glycoprotein. (**F**) Ligand interaction diagram of covalent docking of P1-diacid to cystine 24 on myelin oligodendrocyte glycoprotein. (**G**) Ligand interaction diagram of covalent docking of P2 to cystine 24 on myelin oligodendrocyte glycoprotein. (**H**) Ligand interaction diagram of covalent docking of P2 acid to cystine 24 on myelin oligodendrocyte glycoprotein.

Using the covalent docking tool, the deboronated phosphine-borane compounds and phosphine controls were docked to Cys24 and Cys98 on the MOG monomer 1PKO with the disulfide bond intact. The deboronated phosphine-borane compounds included bis(3-propionic acid methyl ester) phenylphosphine (P1) and (3-propionic acid methyl ester)diphenylphosphine. These compounds contain an ester group that can be hydrolyzed. As such, their metabolized ester bonds form acids. Since P1 has two ester bonds, both an R and S enantiomer exist for P1 (P1-monoacid-R and P1-monoacid-S). As well, a diacid form also exists known as P1-diacid. There is only one ester bond on P2, and when hydrolyzed it is known as P2-acid. Phosphine controls include tris(2-carboxyethyl)phosphine (TCEP) phosphine (P-H), trimethylphosphine (P-Me), and triphenylphosphine (P-Ph). Only isolated phosphine was able to dock to Cys98, and none of the deboronated phosphine-borane compounds studied were able to dock to that cysteine sulfur. In addition, trimethylphosphine, triphenylphosphine, and TCEP were unable to dock to Cys98. All tested compounds were able to dock to Cys24 (see ligand interaction diagrams in Figure 7). All compounds docked with an RMSD less than 1 Å, with negative docking scores and negative molecular mechanics with a generalized Born and surface area solvation (MM-GBSA). The mean docking score of the control compounds was −1.48, compared to −2.06 among the test compounds (*p* = 0.12). The mean MM-GBSA of the controls was −9.10 kcal/mol, compared to −20.83 kcal/mol (*p* = 0.03) (Table 3).

TCEP was the control compound with the most negative docking score (−2.15), and three of the test compounds docked with a more negative docking score than TCEP. Furthermore, all test compounds had an MM-GBSA change in free energy of binding less than −15 kcal/mol, with three test compounds having an MM-GBSA less than −20 kcal/mol. Only isolated phosphine had an MM-GBSA less than −15 kcal/mol, with no other controls meeting this threshold. Of the test compounds, the monoacid metabolite of PB1 in both the S and R enantiomer had the most suitable docking profiles. The S enantiomer had a docking score of −2.46 and an MM-GBSA of −24.99 kcal/mol, and the R enantiomer a docking score of −2.55 and a MM-GBSA of −20.98 kcal/mol. The docking scores and MM-GBSA of the docked compounds to Cys24 in the 1PKO monomer are listed in Table 3.

The amino acids Arg25 and Lys80 were the only amino acids other than Cys24 to have direct interactions with the docked phosphine compounds (Figure 7). Arg25 interacted with all P1 compounds and TCEP. Both the ester groups and carboxylic acid groups of the P1 compounds and the carboxylic acid groups of TCEP interacted with the guanidino group of arginine. Salt bridges were formed between nitrogen and carboxylic acids. Despite the presence of ester groups and carboxylic acid on the P2 compounds, there was no interaction with Arg25. The P2 compounds have an additional phenyl group, a larger effective molecular diameter, and are more rigid than the carboxylic acid chains on P1 compounds. The compounds that interacted with Arg25 had a mean docking score of −2.01 and MM-GBSA of −18.37 kcal/mol, whereas those that did not interact with Arg25 had a docking score of −1.65 and an MM-GBSA of −13.91 kcal/mol.

Lys80 interacted with P1-diacid, P1-monoacid(S), and TCEP through hydrogen bonding and salt bridges. Lys80 was only able to interact with carboxylic acid groups, and not with ester groups. The compounds that interacted with Lys80 has a mean docking score of −2.06 and an MM-GBSA of −14.10 kcal/mol, whereas those that did not had a mean docking score of −1.73 and an MM-GBSA of −17.01 kcal/mol. All tested compounds that interacted with Lys80 also interacted with Arg25.

**Figure 8:**
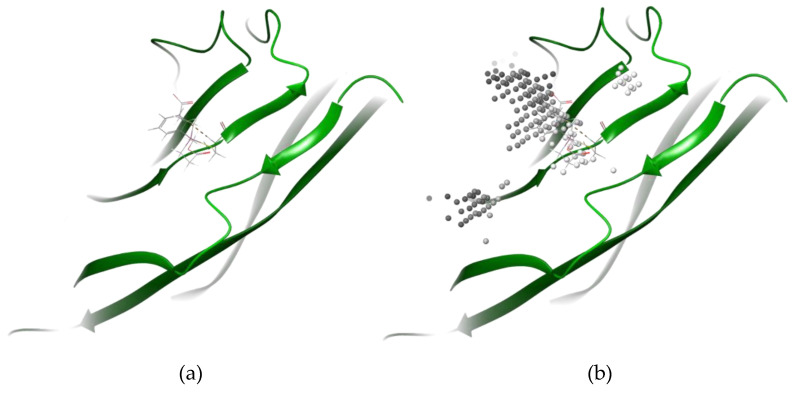
(**a**) Covalent docking results of P1-diacid (ball and stick) in complex with cystine 24 on ribbon diagram of MOG monomer (PBD 1PKO). (**b**) Binding site map on MOG monomer (PBD 1PKO) overlayed with covalent docking results of P1-diacid (ball and stick) in complex with cystine 24 on ribbon diagram of MOG monomer (PBD 1PKO).

To ensure that the Cys24 binding site was accessible, the Schrödinger binding site mapping tool was used to determine which aspects of the protein surfaces were accessible binding sites. These binding site maps were overlapped with the output complexes from the covalent docking procedure to determine if the bound phosphine compounds were docking at a predicted binding site. The binding site mapping tool was first employed without evaluating shallow binding sites, and then with shallow binding sites. Two binding sites were identified when only deep binding sites were evaluated, neither of which included the exposed sulfur of Cys24. However, when shallow binding sites were evaluated, four additional sites were identified. One of these sites included the exposed sulfur on Cys24. This site met all the parameters expected of a valid binding site, with a SiteScore of 0.89, a Dscore of 1.02, and a volume of 421.55 Å. The binding site map over Cys24 in complex with P1-diacid is demonstrated in Figure 8.

### 2.7. Covalent Docking of Phosphine-Borane Compounds to the MOG Dimer Disulfide

The deboronated phosphine-borane compounds and phosphine controls were docked to the MOG-DI dimer. Cys24 and Cys98 in the monomers of the MOG dimer were set as the reactive amino acid and covalently docked, as described previously.

As with the MOG monomer, only phosphine was able to dock to Cys98. None of the deboronated PB1 or PB2 metabolites, trimethylphosphine, triphenylphosphine, or TCEP docked to Cys98. All tested compounds were able to dock to Cys24. All compounds docked with an RMSD less than 1, with negative docking scores and negative prime energies. The mean docking score of the control compounds was −1.25, compared to −2.39 among test compounds (*p* < 0.05), and the mean MM-GBSA of the controls was −11.77 kcal/mol, compared to −21.80 kcal/mol (*p* < 0.01). Of the control compounds, the one with the most negative docking score was isolated phosphine, with a score of −1.55; five of the test compounds were able to dock with a more negative docking score than isolated phosphine. All the test compounds had a prime energy less than −15 kcal/mol, with five having a prime energy less than −20 kcal/mol. Only triphenylphosphine had an MM-GBSA less than −15 kcal/mol, with no other controls meeting this threshold. The docking scores and MM-GBSA for docking of all the studied phosphine compounds to Cys24 in the 1PKO dimer are listed in Table 4.

Similar to the monomer docking results, the amino acids Arg25 and Lys80 both interacted with the docked compounds. However, unlike when docking to the monomer, the amino acid His10 also interacted with docked compounds. Arg25 only interacted through hydrogen bonding and salt bridges with four of the six test compounds and none of the control compounds. Only carboxylic acid groups appeared to interact with the guanidino group of Arg25. All phosphine-borane compounds with a hydrolyzed ester group formed interactions with Arg25. The compounds that interacted with Arg25 had an average docking score of −2.69 and an MM-GBSA of −21.65 kcal/mol, whereas those that did not interact with Arg25 had a docking score of −1.43 and an MM-GBSA of −15.21 kcal/mol.

Interactions of Lys80 were with P1-diacid, P-Ph, and TCEP. The carboxylic acid groups on P1-diacid and TCEP formed hydrogen bonds and salt bridges with the primary amine group on Lys80. The phenyl group of P-Ph formed a Pi-cat interaction with the primary amine group of Lys80. This interaction was not found in any other compound docked. Compounds that interacted with Lys80 had a mean docking score of −1.78 and an MM-GBSA of −17.88 kcal/mol, while those that did not interact with Lys80 had a docking score of −2.00 and an MM-GBSA of −17.75 kcal/mol.

Only P1-monoacid(R) and TCEP interacted with His10. In these interactions, the imidazole ring of His10 formed hydrogen bonds with the carbonyl oxygen of carboxylic acid groups on these compounds. The two compounds that interacted with His10 had a mean docking score of −2.29 and an MM-GBSA of −15.46 kcal/mol, while and those that did not interact with His10 had a docking score of −1.84 and an MM-GBSA of −18.37 kcal/mol.

As with the monomer, the binding site tool was applied to the MOG-DI dimer and then was overlapped with the MOG-DI dimer covalent docking results. A total of six docking sites were identified, of which three were shallow docking sites. One of the shallow binding sites overlapped with the binding site of the phosphine compounds in complex with Cys24. This shallow binding site had a SiteScore of 0.89, a Dscore of 1.02, and a volume of 421.55, and was compatible with a valid binding site.

## 3. Discussion

The molecular modeling demonstrated that the disulfide bond between Cys24 and Cys98 has an impact on the structure of the MOG dimer. Although reducing the disulfide bond made limited structural differences in the MOG monomer, it resulted in a more energetically stable structure. Most of the existing research on MOG evaluates the MOG monomer with the disulfide bond intact. It is unclear if the increased stability in the disulfide-reduced monomer has a clinical or biochemical significance. However, it is possible that favoring a more energetically stable monomer has positive effects, and possibly even relates to the neuroprotection seen with phosphine-borane complexes [8,36]. Cleaving the disulfide bond appeared to impair dimer formation. The total complex energy value of the MOG-DR dimer was nearly 50 kcal/mol lower than the MOG-DI dimer, suggesting that the monomer disulfide bond plays a vital role in dimer formation. Previous studies in computational modeling have suggested that differences between 10 and 30 kcal/mol may suggest greater affinities in docking [37,38]. Furthermore, there was more structural similarity between the MOG-DI dimer and the dimer described by Clements et al. [25] than between the MOG-DR dimer and the Clements et al. dimer. Clements et al. used gel electrophoresis to demonstrate that there is a higher proportion of MOG monomer than MOG dimer [25]. It is possible that reducing environments lead to the cleavage of disulfide bonds and reduced dimer formation, and that in non-pathological states MOG monomers are formed preferentially over MOG homodimers. It has been well known that cleaving disulfide bonds has protective effects on neurons, and one possibility is that this is mediated through preventing the dimer formation of MOG monomers.

The findings in the present study may provide insights into the interaction of phosphine-borane complexes and MOG, with respect to three areas opposing neurodegeneration: remyelination, neurotrophin-related neuroprotection, and the prevention of complement-dependent neuronal loss or synaptic pruning. First, the MOG dimer, as demonstrated previously and replicated in these findings, is an antiparallel head to tail homodimer [25]. This is consistent with the theory that MOG plays a role in adhesion between opposing myelin sheaths [21,25]. Considering that the disulfide bond of the extracellular domain of MOG produces a more stable dimer, it is conceivable that the function of MOG is to promote opposing myelin adhesion in oxidative environments through dimer formation. This may relate to neurodegenerative diseases where oxidative stress plays a role, and by promoting disulfide bond formation (favoring dimer over monomer formation), there may be an increase in opposing myelin sheath adhesion.

There is evidence that myelin adhesion molecules play a role in regulating myelin growth [39]. Elazar et al. found that knockout models of cell adhesion molecules, including Cadm4, Mag, and Caspr, increase the number of axons surrounded by multiple myelin sheaths and identified that in that way neighbouring myelin sheaths overgrew into adjacent territories [39,40]. They suspected that in knockout models, myelin sheath growth may be disinhibited and suggested that cell adhesion molecules play a role in myelin growth regulation [39,40]. This is further supported by studies demonstrating that domains of Cahm4 restricted myelin sheath growth in the CNS and Schwann cell myelination [39,40].

It is possible that MOG dimers behave as a cell adhesion molecule and inhibit myelin sheath growth. In states of oxidative stress, disulfide bond formation (and by extension, dimer formation) may be favored. Such a dimer formation could contribute to the restriction of myelin sheath growth through cell adhesion. Although oxidative stress is associated with neurodegeneration in both the CNS and PNS, there is a stronger implication for oxidative stress playing a role in CNS diseases [41,42,43,44]. This is pertinent, as MOG is absent in the PNS [21,22]. As evidenced by the favorable covalent docking scores that were identified, it is possible that phosphine-borane compounds cleave the disulfide bond of MOG and decrease dimer formation. This may decrease opposing myelin adhesion in oxidate environments and promote myelination and myelin sheath growth.

Second, the reduction of the MOG Cys24-Cys98 disulfide bond may be neuroprotective via the promotion of neurotrophin activity. Büdingen et al. suggested that MOG’s extracellular domain has a similar structure to TrkA. They suggested that MOG binds to nerve growth factor (NGF), the ligand for TrkA, and sequesters NGF from TrkA. They explicitly evaluated the monomer structure of MOG but did not explicitly study the dimer or the impact of the disulfide bond on NGF binding to MOG [45]. It is possible that reducing the disulfide bond of MOG decreases the affinity for NGF, thus sequestering less NGF from TrkA [45,46]. It is therefore possible that phosphine-borane complexes could promote NFG binding to TrkA by reducing MOG’s disulfide bonds.

Third, there is evidence that MOG may act as an activator of the complement cascade by binding to complement component C1q near the IgG binding site [22,47,48,49]. As described by Stevens et al. [50], C1q is elevated in retinal ganglion cells and the inner plexiform layer in glaucoma models compared to controls. It has been suggested that C1q plays a role in synaptic pruning, with C1q knockout models demonstrating a greater number of overlapping projections compared to controls [45]. In a prospective study involving 12 pediatric patients with anti-MOG acquired demyelinating syndromes, 8 were found to have C1q associated to myelin and phagocytic cells in brains with EAE [51]. The studies evaluating MOG–C1q docking did not discuss whether the C1q was binding to MOG as a monomer or as a dimer [22,47,48,52]. However, these studies did prepare their proteins at physiologic conditions where disulfide bonds are formed between Cys24 and Cys98 [25]. Given these findings, it is possible that C1q binds preferentially to the MOG-DI monomer over the MOG-DR monomer. If this is the case, disulfide reduction may be protective from complement binding, and this could explain how phosphine-borane compounds protect neurons from an autoimmune complement cascade.

In all three of the above presented mechanisms, the inhibition of MOG through phosphine-borane compounds would protect retinal ganglion cells from damage. It is therefore possible that phosphine-borane compounds could be used in the treatment of neuro-ophthalmic diseases beyond anti-MOG syndrome.

The major MOG-associated pathophysiology is the anti-MOG antibody syndrome. We found that docking MOG-DI and MOG-DR monomers to the 8-18C5 antibody favored MOG-DI monomer antibody complex formation. This is unsurprising given that the previously determined structure of the MOG antibody complex was between the 8-18C5 antibody and a MOG monomer with the Cys24-Cys98 disulfide bond intact. Our modeling suggests a higher complex energy in the disulfide-reduced structure and increased structural dissimilarity between the MOG-DR monomer complex and the previously determined structure by Breithaupt et al. (2003) [21]. These findings suggest that reducing the MOG disulfide bond could prevent anti-MOG antibody–MOG complex formation. However, there was a lower complex energy between the 8-18C5 antibody and the MOG-DR dimer than the MOG-DI dimer. There has been no previous determination of the MOG dimer–antibody complex, and it is unclear if such a complex is formed. It is possible that if such a complex did exist, then the reduction in the disulfide bond would increase antibody binding. However, given that reducing the disulfide bond decreases dimer formation compared to the MOG-DR monomer, which has decreased antibody binding compared to the disulfide intact structure, it is unclear if the disulfide bond would influence autoimmunity toward the dimer.

In addition, there is more structural similarity between the MOG-DI dimer–antibody complex and the previously determined MOG monomer–antibody complex than the MOG-DR dimer–antibody complex. The structure of the MOG-Fab complex was based on X-ray crystallography of the MOG monomer. It is possible that antibodies preferentially bind MOG monomers vs. MOG dimers [21]. In the previous PBD structure of antibody–MOG complexes (PBD 1PKQ), the disulfide bond on MOG’s extracellular domain was intact. It would be surprising to find that cleaving the disulfide bond would have no impact on antibody–MOG complex formation, given that monoclonal antibodies to MOG are conformation-dependent and antibodies to linear epitopes of MOG do not induce widespread demyelination [35]. It should be noted that the protein quality of the antibody–MOG complex was suitable to analyze the presented structure, but it had insufficient resolution for modeling protein–protein docking. Given the clinical relevance of anti-MOG syndromes, it would be helpful to develop a crystallography structure of an anti-MOG antibody with a resolution and quality more compatible to molecular docking.

It should be noted that dynamic molecular modeling was not employed, with the authors instead using restrained minimization with OLPS3e [53]. The latter approach allowed for a more conservative assessment of the disulfide bond and was more appropriate with the computational power available. However, it should be noted that a dynamic approach could have allowed a possibly wider range of possible structures [54].

The covalent docking results suggested that phosphine-active compounds were able to bind at Cys24 and follow a S_N_2 nucleophilic substitution reaction. The docking scores for all compounds were unimpressive, but these scores typically rely on previous docking patterns. Since this site had never before been targeted for binding and no crystallography of any compound in complex with Cys24 has been determined, these docking scores may be unreliable. However, the MM-GBSAs were highly suggestive that phosphine-borane compound derivatives were compatible with covalent docking to Cys24 in both the monomer and dimer. The site map also provides evidence that the exposed surface of Cys24 was compatible with an acceptable shallow binding site. It should be noted that no negative controls were used in this study. Since there has been no previous crystallography or modeling of this site, there have been no confirmed negative controls for the binding site on the MOG protein. TCEP is known to cleave disulfide bonds indiscriminately and for that reason was used as a positive control [9,10]. Furthermore, the other non PB phosphine molecules all had exposed phosphorus with the ability to complete a S_N_2 nucleophilic substitution reaction with cysteine bonds [10]. However, it should be noted that CovDock in Schrödinger did not dock all phosphine compounds to all disulfide bonds. This is evidenced by the fact that only phosphine was able to cleave Cys98, while all other tested molecules were not able to. This suggests that a negative control would not necessarily be warranted, as CovDock was able to eliminate unsuitable targets.

These findings suggest that Cys24’s disulfide bond could be an appropriate target for drug discovery with respect to anti-MOG syndrome. Salt bridge and hydrogen bond formation with Arg25 had a significantly positive effect on docking. The development of phosphine-borane compounds that could more specifically interact with Arg25 could help create a MOG-targeted reducing agent. In particular, P1 and its metabolites had favorable MM-GBSAs. This may indicate that the targeted hydrolysis of phosphine-borane ester bonds may improve the binding of phosphine-borane to the MOG Cys24-Cys98 disulfide bond because carboxylic acid groups are more likely to make hydrogen bonds with the amino acid than the intact ester group.

It is important to note that this study did not involve in vivo or in vitro methodologies. The isolation of MOG and anti-MOG antibodies is both a challenging and costly process, and there have been relatively few studies assessing ligand docking to these proteins. The current in silico evaluation was necessary to identify potential ligands and protein binding sites, providing a preliminary proof of concept. Before these molecules can be considered for clinical trials, comprehensive in vivo and in vitro studies will be required.

Patients with anti-MOG syndrome are often subjected to long-term immunosuppression and the potential adverse effects of these medications [11,18]. Unlike systemic immunosuppression, a phosphine-borane compound could theoretically be synthesized that would be specific to the MOG protein, thereby potentially mitigating any adverse effects.

## 4. Materials and Methods

### 4.1. Compounds

**Figure 9:**
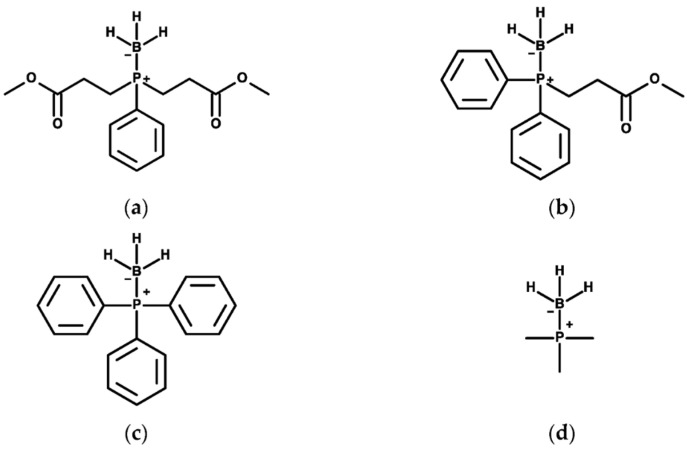
Phosphine-borane structures. Chemical structures of (**a**) PB1, (**b**) PB2 (**c**) PB-Ph, and (**d**) PB-Me.

Phosphine-borane compounds were synthesized at the Keck–University of Wisconsin Comprehensive Cancer Center Small Molecule Screening Facility (Madison, WI). PB1 and the (3-propionic acid methyl ester) diphenylphosphine–borane complex PB2, and their deprotected metabolites bis(3-propionic acid methyl ester) phenylphosphine (P1) and (3-propionic acid methyl ester)diphenylphosphine (P2), cleave intracellular disulfide reporters and protect RGCs from oxidative stress. The structures of select structures are presented in Figure 9. These compounds have ester bonds (two in P1 and one in P2) that can be hydrolyzed, as previously identified by in vivo NMR [7,8,9], resulting in six P1 and P2 compounds with exposed phosphorus. These compounds were used as test compounds for molecular docking. Given that the disulfide bond on MOG has never been targeted previously, positive controls for its specific reduction could not be used. Instead, tris(2-carboxyethyl)phosphine (TCEP), a reducing agent known to cleave generic disulfide bonds, was used. TCEP, like phosphine-borane compounds, cleaves disulfide bonds through an S_N_2 nucleophilic substitution reaction, with the exposed phosphorus acting as a nucleophile [10]. Phosphine (PH_3_), trimethylphosphine (P(CH_3_)_3_), and triphenylphosphine (P(C_6_H_5_)_3_) all have exposed phosphorus and could potentially cleave MOG’s solitary disulfide bond. TCEP, phosphine, trimethylphosphine, and triphenylphosphine therefore served as theoretical positive controls for molecular docking.

### 4.2. Protein Structures

The extracellular structure domain of the monomer and dimer of MOG was described by Clements et al. [25]. The monomeric structure of MOG (PBD 1PKO) was also generated through X-ray diffraction at 1.4 Å resolution, R-value free of 0.22, R-value work of 0.19, and total atom count of 1160) [21]. The latter structure was imported into Schrödinger 2021-1 (Schrödinger LLC, New York, NY, USA; version 12.6.144) and prepared using the Protein Preparation wizard [55].Bond orders were assigned using the CCD database. All missing hydrogens were added, missing side chains were added using prime and het, and stats were generated using the Epik function of Schrödinger at pH 7 ± 2. Hydrogen bond assignment was optimized from sample water orientations at pH 7. Water molecules more than 5 Å from het groups were removed, as well as those with less than 3 hydrogen bonds to non-water molecules. Finally, restrained minimization was performed on all atoms within the structure using the OLPS3e force field, to determine the lowest energy structure to an RMSD of 0.3 Å [53]. The result of these steps was the MOG monomer with the disulfide bond intact (“MOG-DI monomer”).

The disulfide-reduced monomer of MOG was created by identifying the disulfide bond on MOG through the Schrödinger Maestro platform, and then the disulfide bond between Cys24 and Cys98 was manually cleaved. The post-cleaved structure was processed using the Protein Preparation wizard as described previously, but without allowing the creation of disulfide bonds. The result was the MOG monomer with the disulfide reduced (“MOG-DR monomer”).

Breithaupt et al. (2003) also generated a PBD structure (1PKQ; resolution 3 Å, R-value free 0.32, R-value work 0.25, total atom count 8517) of the extracellular domain of MOG in the Fab-complex, for the purposes of understanding MOG’s role in autoimmunity [21]. This was achieved by determining the complex between MOG’s extracellular domain and the anti-MOG antibody 8-18C5. This structure was imported into Schrödinger and prepared using the Protein Preparation wizard, as described above. The structure included two MOG monomers and two antibodies, with both heavy and light chains. We separated and isolated antibodies and MOG monomers into separate entities in Schrödinger. The isolated antibodies and MOG monomers were then prepared using the Protein Preparation wizard, as described above.

### 4.3. Protein Docking

Using the Schrödinger protein–protein docking tool, the MOG-DI and DR monomers were docked in order to determine the structure of the MOG-DI and DR homodimers. This was accomplished using the dimer mode in the Prime Protein–Protein docking function. The docking tool available in Schrödinger has been previously validated in vivo, allowing for a suitable evaluation of the protein modifications [56,57]. Amino acid constraints were not labeled as attractive or repulsive. However, the monomer tail (AA 118–126) was assigned the buried state because these amino acids do not form the primary extracellular structure, are not involved in the dimer interface, and are located nearest to the myelin interface. Setting amino acids as buried results in increased van der Waals radii and repulsive potentials for buried residues and sets the attractive potential for residues to zero. The number of protein orientations to dock was set to the maximum-allowed 70,000 orientations. This represents a sampling of every 5° in the space of Euler angles. The number of poses to return was set to 30, so that only the highest-scoring 30 poses would be evaluated. The most energetically favorable pose of each complex was used for comparative analysis. Protein energetics was measured using total complex energy in kcal/mol. Total complex energies were determined by the determination of bond and molecular mechanism force fields through electrostatic and van der Waals energies and solvation free energy contributions through polar electrostatic energies and the nonelectrostatic solvation component, as implemented in the Schrödinger protein–protein docking tool [57,58].

Using the Schrödinger protein-protein docking tool, the MOG antibody complex was generated against the MOG-DI monomer, MOG-DR monomer, MOG-DI dimer, and MOG-DR dimer. The 8-18C5 anti-MOG antibody was isolated from 1PKQ, as described above. No amino acids were constrained as attraction, repulsion, or buried. The heavy chain of the antibody was detected using the default system in the Schrödinger protein–protein docking tool available in Schrödinger. The number of orientations was set to 70,000 and the number of poses to 30. The protein metrics evaluated prior to protein docking included PBD resolution, binding sites, bond angle length and angles, protein backbone and sidechain structure steric clashes, and protein site maps [59]. Given that MOG is a membrane-bound protein and approaches of the antibody or MOG monomer from the membrane side could not be completely eliminated with constraints, the protein chosen for analysis was the one with the most structural similarity to the structures presented in Clements et al. and Breithaupt et al. [21,25] Such a methodology makes fewer assumptions regarding the final protein structure than more liberal approaches [60]. These methodologies have been previously validated and compared to other in silico models in syntaxin superfamily proteins and peptide ligand complexes [61,62].

To analyze the interface of the docked proteins, ProFunc was employed to evaluate the number of residues involved at the interface, the area of the interface, the number of salt bridges at the interface, and the number of hydrogen bonds at the interface [55].

### 4.4. Covalent Docking

Using the Covalent Docking (CovDock) feature of Schrödinger, all compounds described above were covalently docked to Cys24 and Cys98 in the MOG-DI monomer and MOG-DI dimer. CovDock has been effectively demonstrated to exhibit consistency with in vivo modes of interaction, indicating its reliability and relevance in predicting molecular interactions, with the CovDock RMSD ranging between 1.5 and 1.9 Å [63,64,65,66,67]. To simulate the S_N_2 nucleophilic substitution reaction [10], the following code was used to describe this reaction:
**REACTION_NAME****CUSTOM CODE**CUSTOM_CHEMISTRY(‘<1>’, (‘charge’, 0, 1))CUSTOM_CHEMISTRY(‘<1>|<2>’, (‘bond’, 1, (1, 2)))CUSTOM_CHEMISTRY(‘<2>’, (‘charge’, 1, 1))LIGAND_SMARTS_PATTERN1,[PX4+1,PX3+0]RECEPTOR_SMARTS_PATTERN2,[C,c]-[S,O;H1,-1]

Docked ligands were confined to enclosing boxes centered around the reactive residue of interest (either Cys24 or Cys98). No constraints were applied to any amino side chain. Docking was completed with the Schrödinger thorough pose prediction methodology. Molecular mechanics energies combined with a generalized Born and surface area continuum solvation (MM-GBSA) scoring and docking scores were calculated as the primary metrics of the docking results. MM-GBSA scoring is a force field-based assessment of docking with the calculated binding energy dependant on force field predictions, charge models, continuum solvation methods, sampling methods, interior dielectric constants, and conformation entropy. Such scoring methodologies have become accepted practice as they demonstrate high accuracy and are less computationally demanding than other force field-based calculations [67]. The docking scoring in Schrödinger is calculated empirically based on lipophilic–lipophilic terms, hydrogen bond terms, a rotatable bond penalty, contributions from protein–ligand coulomb-vdW energies, and hydrophobic encounters [68,69]. The most energetically favorable pose of each compound was used for comparative analysis. The RMSD was determined for all molecular docking results as a metric for validity and metric for the reproduction of findings. The RMSD is calculated by comparing the multiple binding poses to each other [70]. The metrics evaluated in covalent docking included evaluating the structure of the ligand and protein at the site map as well as electrostatic, lipophilic, and aromatic interactions, H-bonds, rotational entropy, ligand flexibility, and the stability of the produced covalent bond [66,71].

### 4.5. Site Map Determination

To determine if the site of the compound binding was compatible with an appropriate receptor site, the SiteMap tool in Schrödinger was employed to determine the top ranked potential receptor binding sites. A minimum of 15 site points were required in order to be considered a site. The most restrictive definition of hydrophobicity was used, and sites less than 4 Å from the nearest site point were combined into a single site. A site map was determined for both the MOG-DI monomer and MOG-DI dimer. SiteMap was run twice for each structure, first without allowing for the detection of shallow binding sites and then with the detection of shallow binding sites.

## 5. Conclusions

Based on the in silico modeling presented above, we suggest that the Cys24-Cys98 disulfide bond in the extracellular domain of MOG could play an important role in MOG dimer formation. It is plausible that the neuroprotective effects of disulfide-cleaving phosphine-borane compounds are mediated by preventing dimer formation, which subsequently regulates myelin formation, the sequestration of nerve growth factor, and the activation of autoimmune complement cascades. Disruption of the Cys24-Cys98 bond also reduces the stability of anti-MOG antibody complexes, providing a potential avenue to treat anti-MOG-related disease. Although the presented computational modeling is promising, it should be noted that experimental validation is required. If verified, phosphine-borane compounds may provide clinicians with a new approach to treating one of the most serious optic neuropathies.

## Figures and Tables

**Table 1:** MOG structures with total complex energy values in both disulfide bond intact and disulfide bond reduced structures.

MOG Structures	Total Complex Energy Values (kcal/mol)	
	Disulfide Bond Intact	Disulfide Bond Reduced
Monomer	−527.04	−582.26
Dimer	−1218.89	−1172.76
Monomer antibody complex	−1332.73	−1333.79
Dimer antibody complex	−2029.64	−2032.65

**Table 2:** Summary of protein interaction interfaces between the disulfide intact and reduced structures generated by ProFunc.

		Interface Residuals	Interface Area (Å^2^)	Salt Bridges	Hydrogen Bonds	Nonbond Contacts
Dimer	Intact	57	2060	8	15	174
	Reduced	42	2020	-	6	177
Monomer antibody complex	Intact	24	1169	3	9	106
	Reduced	21	1116	3	5	74
Dimer antibody complex	Intact	23	1356	3	7	76
	Reduced	34	1609	1	7	134

**Table 3:** Covalent docking results of phosphine compounds to cystine 24 on myelin oligodendrocyte protein monomer.

	CovDock Score	MM-GSBA
P1	−1.30	−28.54
P1-Monoacid-R	−2.55	−20.98
P1-Monoacid-S	−2.47	−24.99
P1-Diacid	−1.59	−15.72
P2	−1.98	−18.61
P2-Acid	−2.49	−16.16
P-Ph	−1.35	−21.88
P-Me	−0.90	−7.96
P-H	−1.53	−4.96
TCEP	−2.15	−1.60

**Table 4:** Covalent docking results of phosphine compounds to cystine 24 on myelin oligodendrocyte protein disulfide intact dimer.

	CovDock Score	MM-GSBA
P1	−1.22	−24.97
P1-Monoacid-R	−2.37	−22.45
P1-Monoacid-S	−3.11	−20.80
P1-Diacid	−2.48	−21.60
P2	−2.34	−19.21
P2-Acid	−2.82	−21.76
P-Ph	−1.37	−21.93
P-Me	−0.61	−9.71
P-H	−1.55	−5.34
TCEP	−1.48	−10.11

## Data Availability

The data presented in this study are available within the article.
